# A Comparison Between Ultrasound-Guided Supraclavicular and Infraclavicular Approaches to Brachial Plexus Block for Elective Upper Limb Surgery

**DOI:** 10.7759/cureus.46656

**Published:** 2023-10-07

**Authors:** Avinash Guru, Dilip Chandar Desingh, Vigneswaran Jayakumar, Suresh Kumar Kuppusamy

**Affiliations:** 1 Anaesthesiology, Dhanalakshmi Srinivasan Medical College and Hospital, Perambalur, IND; 2 Anaesthesiology, Sri Manakula Vinayagar Medical College and Hospital, Pondicherry, IND

**Keywords:** inplane approach, 0.25% bupivacaine, supraclavicular brachial plexus block, ultra sound guided brachial plexus nerve block, postoperative analgesia, upper limb surgery, ultrasound, supra clavicular block, infraclavicular block, regional anaesthesia

## Abstract

Background: Regional anaesthesia offers the anaesthesiologist, the surgeon, as well as the patient advantages over general anaesthesia such as being conscious through the surgery, avoiding multiple drugs, better haemodynamic stability, excellent postoperative analgesia, and faster per oral consumption post surgery. Compared with the axillary approach, the brachial plexus block at the level of the clavicle can anaesthetize all four distal upper extremity nerve territories without the requirement for a separate block of the musculocutaneous nerve.

Aim: The aim of the study was to compare the effect of both supraclavicular and infraclavicular brachial plexus blocks in terms of time taken for onset, performance, and block success.

Materials and methods: Sixty patients undergoing below-elbow upper limb surgeries were randomized into two groups: (i) supraclavicular (Group S) and (ii) infraclavicular (Group I). All patients received 30ml 0f 0.5% bupivacaine as the local anesthetic of choice. The block performance time, time taken for onset of sensory and motor blockade, total duration of block, and hemodynamic parameters were observed. The block performance times and the onset of the sensory blockade were the primary outcomes while the duration of the block and hemodynamic parameters were secondary outcomes. Two two-tailed independent sample t-tests will be used to compare the variables.

Results: We observed that the block performance time for the infraclavicular block (mean 14.833 minutes) was longer than the supraclavicular block (mean 10.37 minutes). This was statistically significant with p <0.001. In terms of onset of sensory blockade, the infraclavicular group (13.667 minutes) had a quicker onset compared to the supraclavicular group (17.333 minutes). This was also statistically significant with p <0.001. The mean total duration of sensory and motor blockade was similar in both groups (p-value of 0.341 and 0.791 respectively) and there was no statistical difference. There was no hemodynamic instability or complications in our study.

Conclusion: Ultrasound-guided infraclavicular block is a relatively safer technique when compared to the supraclavicular technique with faster onset. The time taken for administering the infraclavicular block can be reduced by repeated exposure to the technique.

## Introduction

The majority of the surgeries involving the upper limb are done under regional anaesthesia by instilling local anaesthetics around the brachial plexus. The first documented brachial plexus was done by William Stewart Halsted in the late 19th century. Over the years, the technique has evolved from a crude anatomical landmark-guided technique to precise injection of the drugs at the target site by means of using an ultrasound. Both supraclavicular and infraclavicular brachial plexus blocks can be performed for upper limb surgeries, but anaesthesiologists tend to have a predilection towards the predecessor over the infraclavicular approach for brachial plexus block due to the perplexity of technique and a higher rate of complications while performing a blind infraclavicular approach [1-3].

With the advent of ultrasonography, peripheral nerve blocks for regional anaesthesia have become less cumbersome. Ultrasound-guided regional anaesthesia (UGRA) offers a real-time visualization of anatomic structures in relation to the nerve bundles and with the advent of echogenic needles, the needle tip and drug spread can be easily visualized making UGRA a preferred technique compared to its traditional counterparts. We proposed that the infraclavicular approach of brachial plexus block would be quicker to perform and relatively safer than the supraclavicular approach, with fewer complications when performed under ultrasound guidance [1,4-16].

## Materials and methods

This prospective, single-blinded, randomized control study was done in a tertiary teaching hospital in South India from October 2017 to May 2019. The Institutional Ethics Committee of Sri Manakula Vinayagar Medical College and Hospital approved the study (approval number: SMVMCH-EC/DO/AL/1261/2017) and the study was registered under the Clinical Trial Registry of India (registration number: CTRI/2019/05/018969). The primary objective of the study was to compare the ease in administering supraclavicular and infraclavicular brachial plexus blocks, respectively, in terms of time taken for onset, performance and block success, and the secondary objective was to determine which approach produced better success with the least complications.

The sample size was calculated with the help of OpenEpi version 3.0 and with a mean Visual Analogue Score between two groups of supraclavicular and infraclavicular blocks each with a mean time of block of 17(±9) and 10(± 7), respectively, from a previous study by Roussel et al. [8]. With a 95% confidence interval (CI), 10% non-response and 80% power, and 1:1 allocation, we got a sample population of 30 in each group, making a total of 60 participants.

Sixty patients aged between 18-65 years belonging to the American Society of Anesthesiologists (ASA) physical status I and II scheduled to undergo elective below elbow upper limb surgeries were enrolled in the study. Patients who had contraindications to regional anaesthesia, pregnant females, and patients with mental incapacity or language barrier, precluding informed consent were excluded from study. The nature of study was explained to all the study participants and a written consent was obtained from them. The study participants were randomized into two groups (Group I and Group S) by block randomization technique. The randomization sequence was generated by the epidemiology unit of the institute. There were a total of 10 blocks, each consisting of six patients. After obtaining consent, the principal investigator (based on randomization sequence) disclosed the approach of brachial plexus block to the attending anesthesiologist. The patients belonging to Group I received an infraclavicular block while that of Group S received a supraclavicular block. Both groups received 30ml of 0.5% bupivacaine as the local anesthetic for brachial plexus block.

All the study participants were premedicated and kept on fasting for eight hours following institutional anaesthesia protocol. The patients were shifted to the operation theatre and routine monitors including ECG, saturation of peripheral oxygen (SPO2), and non-invasive blood pressure (NIBP) was attached to the patients following basic standards for monitoring recommended by ASA. The brachial plexus blocks were administered using a linear ultrasound probe (SonoSite® 7-12 MHz linear array transducer; Fujifilm, Bothell, Washington, United States) by an experienced anesthesiologist. For the supraclavicular brachial plexus block, the ultrasound probe was placed in the frontal plane in the fossa above the clavicle and a short axis view of the subclavian artery was obtained [10]. The needle was then advanced in the plane with direct visualization from the lateral aspect until the tip of the needle was located closer to the subclavian artery. After thorough negative aspiration, the drug was injected into the brachial plexus along with slight manipulation of the needle tip ensuring proper spread of the drug among all compartments of the plexus.

In the infraclavicular brachial plexus block, the ultrasound probe was placed in the para-sagittal plane inferior to the clavicle obtaining a short axis view of the axillary artery. The needle was inserted and advanced in-plane technique until the tip of the needle was located just posterior to the axillary artery in the midst of chords of the brachial plexus [10]. After confirmation of the appropriate needle tip position and negative aspiration, the local anesthetic was administered.

The time taken for onset and degree of sensory and motor block was assessed every fifth minute for 30 minutes until complete block was achieved. If complete sensory blockade was not achieved even after 30 minutes and the patient perceived pain, it was taken as a block failure. A rescue block at an appropriate level was given if a single nerve was spared. In the unfortunate event of sparing more than one nerve, general anaesthesia was planned to be administered. Response to pinprick was used to assess sensory block. The areas supplied by median, radial, ulnar, musculocutaneous, and medial cutaneous nerves of the forearm were assessed individually and noted. The scoring system adapted from Koscielniak-Nielsen et al. [3] (0 - sharp pain, 1 - touch sensation only, and 2 - no sensation) was used for checking sensory block. A score of 2 was taken as the end point for the onset of sensory blockade.

**Figure 1:**
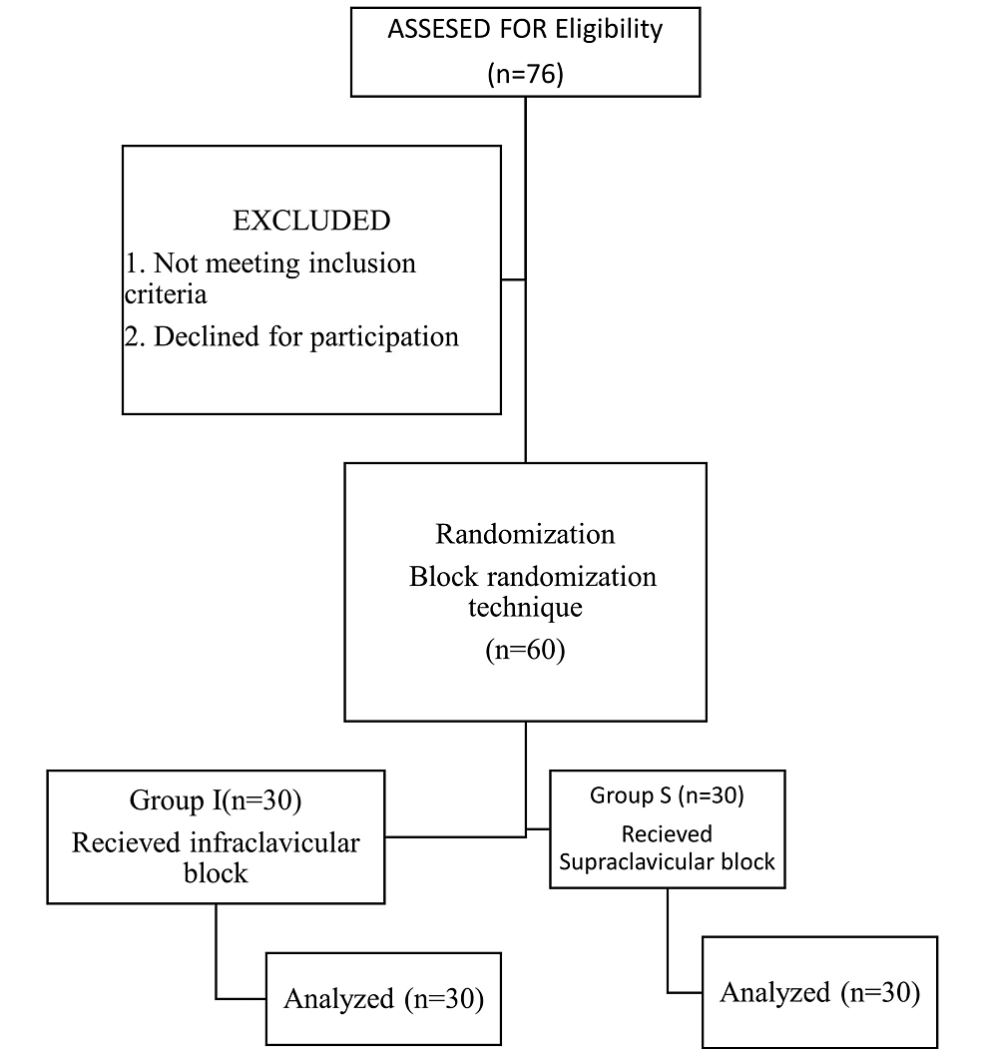
Consolidated Standards of Reporting Trials (CONSORT) chart of patients

Statistical analysis

The collected data was entered on a Microsoft Excel worksheet (Microsoft Corporation, Redmond, Washington, United States). the data analysis was done using IBM SPSS Statistics for Windows, Version 21.0 (Released2012; IBM Corp., Armonk, New York, United States). The study variables were expressed as mean and standard deviation. Two-tailed independent sample t-test was used to compare the variables. and a p-value less than 0.05 was considered significant.

Motor blockade was assessed simultaneously according to the modified Lovett scale, in the four nerve areas: thumb abduction for the radial nerve, thumb adduction for the ulnar nerve, thumb opposition for the median nerve, and flexion of the elbow for the musculocutaneous nerve. The time of onset of motor blockade and the time of return of normal movement were noted. The duration of the motor blockade was considered from the onset to the return of normal movement. The time taken from the positioning of an ultrasound probe to the removal of the needle after injecting the local anesthetic was taken as block performance time. The time at which the patient had no sensation to pinprick was noted as the time taken for a complete sensory blockade and had no movement of the upper limb below the elbow was taken as the time taken for a complete motor blockade.

## Results

The observations are displayed as tables and line graphs for interpretation convenience. Table 1 shows the age and sex distribution between both groups and they were comparable (p=0.792).

**Table 1:** Age and sex distribution between the study groups

		Type of block		p value
		Infraclavicular, n (%)	Supraclavicular, n (%)	
Age in Years	18-30	8 (26.7)	6 (20.0)	0.792
	31-45	10 (33.3)	12 (40.0)	
	46 and above	12 (40.0)	12 (40.0)	
Sex	Male	21 (70.0)	15 (50.0)	0.114
	Female	9 (30.0)	15 (50.0)	

Table 2 Shows the body mass index (BMI) distribution between the groups and the groups were comparable. 

**Table 2:** Body mass index (BMI) distribution between the groups

BMI category	Type of block		p-value
	Infraclavicular, n (%)	Supraclavicular, n (%)	
Normal	20 (66.7)	12 (40.0)	0.068
Overweight	10 (33.3)	16 (53.3)	
Obese	0	2 (6.7)	

The block performance time (Table 3) was relatively quicker in the supraclavicular group (10.31 ± 1.52 minutes) than in the infraclavicular group (14.83 ± 1.45 minutes) and it was statistically significant (p< 0.001). The onset of sensory blockade was early (13.67 ± 22.25 minutes) in the infraclavicular group compared to the supraclavicular group (17.33 ± 2.54 minutes) and was found to be statistically significant (p< 0.001). However, there was no significant difference in the duration of sensory block in both groups (p= 0.341). The onset of motor blockade was similar in both groups. Even the duration of the motor block didn’t have any significant difference (p= 0.791) between the groups.

**Table 3:** The block parameters during the study

Parameter	Type of block		p-value
	Infraclavicular, Mean (SD)	Supraclavicular, Mean (SD)	
Block performance time (minutes)	14.83 (1.45)	10.31 (1.52)	<0.001
Onset of sensory block (minutes)	13.67 (2.25)	17.33 (2.54)	<0.001
Duration of sensory block (minutes)	602.33 (79.31)	583.17 (75.32)	0.341
Rescue analgesia (minutes)	602.33 (79.31)	583.17 (75.32)	0.341
Onset of motor block (minutes)	18.50 (2.33)	18.50 (2.33)	0.999
Duration of motor block (minutes)	537.17 (51.74)	540.83 (54.96)	0.791

**Figure 2:**
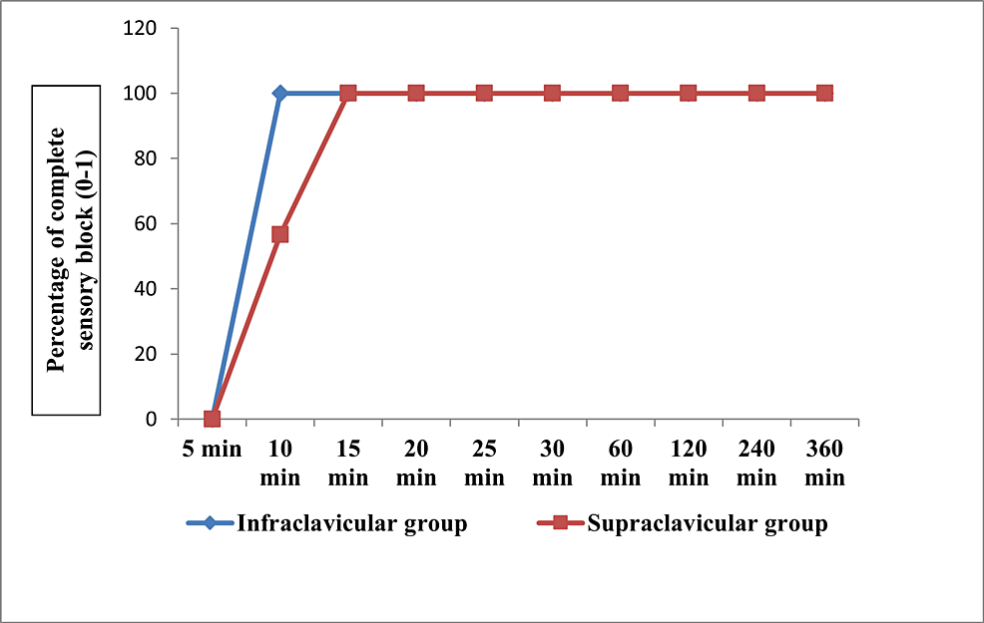
Time taken to achieve complete sensory block in the four dermatomes between the groups in terms of proportion (N= 60)

**Figure 3:**
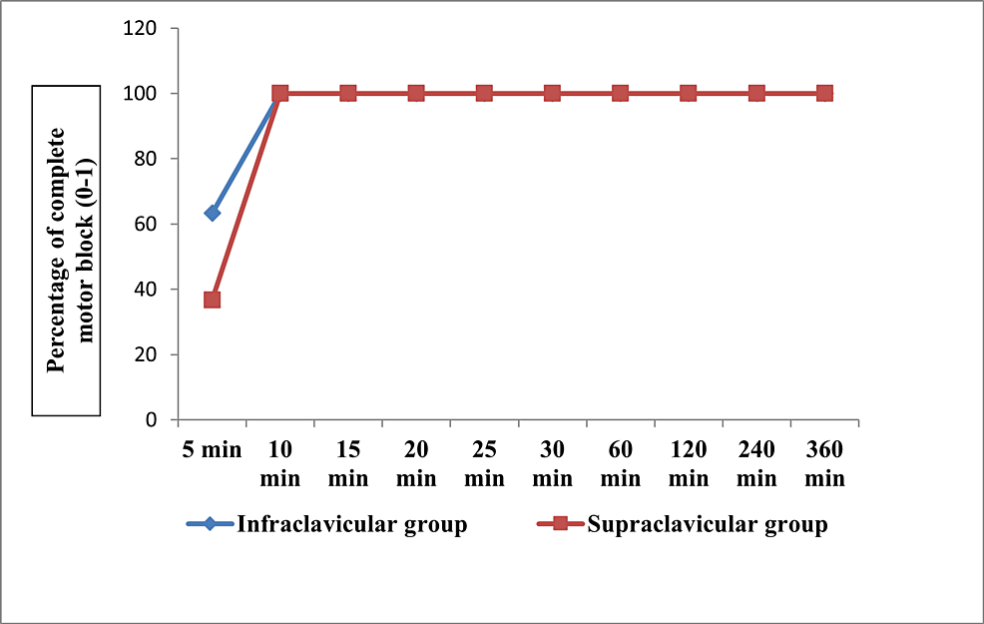
Time taken to achieve complete motor block in four nerves between the groups in terms of proportions

Figures 2-3 show the progression of sensory and motor blockade of individual nerve dermatomes at various time intervals between the groups. There were no differences in the progression of the sensory block. However, there was a significant difference at 10 minutes and 15 minutes between the two groups (p<0.001).

## Discussion

Regional anaesthesia is used widely nowadays, owing to its multiple advantages when compared to general anaesthesia. There is increasing evidence showing that regional anaesthesia produces a good reduction in postoperative pain, side effects, and intraoperative and postoperative morbidity, which in turn results in shorter stays in postoperative anaesthesia care [1,3-5]. In our study, a comparison of the supraclavicular and infraclavicular approach to the brachial plexus was carried out, which indeed is one of the fewer studies conducted in this part of the country to decipher and compare the effects of infraclavicular and supraclavicular blocks.

In the present study, the age, gender and BMI categories were comparable across the groups and, hence, it can be accounted that the confounding effect of these variables on the outcome was kept to the minimum. Both the groups received the blocks under ultrasound guidance, participants in neither group required supplementary rescue blocks or conversion to general anaesthesia. Complications like pneumothorax and nerve injury-related paresthesia were not reported in the study participants.

The block performance time for the supraclavicular block was less compared to the infraclavicular block. This finding contrasts with the studies conducted by Abhinaya et al., Koscielniak-Nielsen et al., and Gurkan et al., in which they reported that block performance time was less in the infraclavicular block when compared to the supraclavicular block. However, a study conducted by Sarkar et al. reported similar findings as our study [1,3-5]. This might be due to the unfamiliarity of the methodology and relative technical difficulty in performing infraclavicular brachial plexus block. Studies conducted by Satani et al., Park et al., Arcand et al., and Yang et al. reported that there was no difference in block performance time between the two types of block [6-9]. But the majority of the above studies were done using a landmark-guided technique or a nerve stimulator technique and it would be unwise to generalize the findings for UGRA.

The onset of motor block was similar in both groups, whereas the onset of sensory blockade was earlier in infraclavicular block (13.67 vs 17.33 minutes) as compared to supraclavicular block and it was found to be statistically significant (p<0.001). The same pool of evidence was also generated from the study conducted by Abhinaya et al. and Koscielniak-Nielsen et al., in which the onset of sensory blockade was faster in infraclavicular block [1,3]. These findings support the hypothesis that an infraclavicular block is safe and can be performed faster than a supraclavicular block in the presence of ultrasound [1].

Motor blockade was also assessed simultaneously according to the modified Lovett scale, in the four nerve areas. At five minutes, a motor blockade was achieved earlier in the infraclavicular group for all the motor nerves studied and it was statistically significant. These findings may be attributed to the traditional mantle and core hypothesis in a local anesthetic spread in regional anaesthesia [17]. In the upper limb neuraxial anatomy, the neurons supplying the distal part of the upper limb are located more in the centre of the nerve compared to the nerves supplying the proximal areas. The level of block in the supraclavicular approach is comparatively high in the brachial plexus anatomy; hence, there is a delay in diffusion of local anesthetic for the neurons supplying below elbow level. These results were similar to the study carried out by Yang et al. [2]. However, there was no difference in the duration of the blockade, both motor and sensory blockade, which is similar to studies conducted by Abhinaya et al., Satani et al., Bharti et al., and Roussel et al. [1,6-10].

The block performance time in the infraclavicular group in our study was longer when compared to the supraclavicular group. The plexus is situated at a deeper plane in the infraclavicular approach; also, the angle of approach is very narrow, which results in technical difficulty. Obesity also adds to the technical difficulty in the infraclavicular approach. Infrequent practice might also be a reason that influenced the longer duration for performing the infraclavicular block. However, with a high success rate and no reported complications observed in our study, more frequent use of the infraclavicular approach might eventually result in similar block performance times to that of the supraclavicular approach or even less.

The major advantage of the study is that, to the best of our knowledge, it is the first of its kind in this part of the country that involved proper randomization of the participants; thereby, the chance of selection bias was kept to a minimum. The limitation of the study is the small sample size. A larger sample size would have resulted in more power which could have added more strength to the study; however, this was practically difficult owing to time constraints.

## Conclusions

Ultrasound-guided Infraclavicular block is a relatively safer technique for anaesthetizing upper limb surgeries. The evolution of motor and sensory blockade was better for the infraclavicular block when compared to the supraclavicular block. Block performance time for the supraclavicular approach of the brachial plexus block was shorter than that of the infraclavicular approach of the brachial plexus block. The time lag that occurs while administering the infraclavicular block can be circumvented by repeatedly using the infraclavicular technique of brachial plexus block for upper limb surgeries. 
